# Seroprevalence of SARS-CoV-2–Specific Antibodies among Quarantined Close Contacts of COVID-19 Patients, Faroe Islands, 2020

**DOI:** 10.3201/eid2711.204948

**Published:** 2021-11

**Authors:** Maria Skaalum Petersen, Marnar Fríðheim Kristiansen, Halla Weihe Reinert, Jógvan Páll Fjallsbak, Debes Hammershaimb Christiansen, Shahin Gaini, Bjarni á Steig, Lars Fodgaard Møller, Marin Strøm, Pál Weihe

**Affiliations:** University of the Faroe Islands, Tórshavn, Faroe Islands (M.S. Petersen, M.F. Kristiansen, S. Gaini, M. Strøm, P. Weihe);; The Faroese Hospital System, Tórshavn (M.S. Petersen, H.W. Reinert, P. Weihe);; National Hospital of the Faroe Islands, Tórshavn (M.F. Kristiansen, S. Gaini, B. á Steig);; COVID-19 Task Force, Ministry of Health, Tórshavn (M.F. Kristiansen, B. á Steig);; Faroese Food and Veterinary Authority, Tórshavn (J.P. Fjallsbak, D.H. Christiansen);; Odense University Hospital, Odense, Denmark (S. Gaini);; University of Southern Denmark, Odense (S. Gaini); Chief Medical Officer Office, Tórshavn (L.F. Møller);; Statens Serum Institut, Copenhagen, Denmark (M. Strøm)

**Keywords:** SARS-CoV-2 seroprevalence, close contacts, Faroe Islands, secondary attack rate, SARS-CoV-2, COVID-19, respiratory infections, severe acute respiratory syndrome coronavirus 2, 2019 novel coronavirus disease, coronavirus disease, zoonoses, viruses, coronaviruses, quarantine, seroprevalence, antibodies

## Abstract

Close contacts of coronavirus disease (COVID-19) patients are at high risk for severe acute respiratory syndrome 2 (SARS-CoV-2) infection. We assessed the seroprevalence of SARS-CoV-2–specific antibodies among quarantined close contacts of COVID-19 patients in the Faroe Islands. We invited quarantined close contacts of COVID-19 index patients identified during March 3–April 22, 2020, to participate in this study; 584 (81%) contacts consented and underwent serologic testing. Among the 584 participants, 32 (5.5%) were seropositive for total antibody against SARS-CoV-2. Household and young or elderly contacts had higher risk for seropositivity than other contacts. We found a secondary attack rate of 19.2%. Seroprevalence among close contacts was almost 10-fold higher than among the general population of the Faroe Islands. Regularly testing household close contacts of COVID-19 patients might help track the transmission of SARS-CoV-2.

Reverse transcription PCR (RT-PCR) is a standard tool for diagnosing severe acute respiratory syndrome coronavirus 2 (SARS-CoV-2) infection. However, different testing strategies might cause wide variation in the number of identified subclinical and asymptomatic cases, which could remain undetected (*1*). In May 2020, the Faroe Islands, an autonomous country that is part of the kingdom of Denmark with a population of 52,554 persons, had a 0.6% seroprevalence of antibodies against SARS-CoV-2 (*2*), among the lowest reported seroprevalences worldwide (*3*,*4*). This low seroprevalence is probably influenced by large-scale testing in the Faroes (*5*,*6*). However, the study also identified a few previously undetected cases, implying that persons had been infected without knowing and without spreading the contagion (*2*).

Since the identification of the first imported case of coronavirus disease (COVID-19) in the Faroe Islands on March 3, 2020, the territory has complied with World Health Organization recommendations to use an active suppression strategy focusing on testing and isolating patients and their close contacts. Accordingly, all close contacts of COVID-19 patients in the Faroe Islands were advised to quarantine for 2 weeks (*5*). In the Faroe Islands, the first wave of the COVID-19 pandemic ended on April 22, 2020; no local cases were detected until August 3, 2020, when a second surge began (*6*). During the first wave, the Faroe Islands’ per capita testing rates were among the highest in the world. The seroprevalence study showed that, perhaps because of low levels of community transmission, few cases remained undetected (*2*). As a result, this context provides a unique opportunity to investigate the transmission dynamics of SARS-CoV-2 using serologic tests.

SARS-CoV-2 is highly contagious. Family members and other close contacts of COVID-19 patients are at higher risk for SARS-CoV-2 infection, potentially furthering the transmission of disease (*7*). Most studies estimating the secondary attack rate of SARS-CoV-2 use RT-PCR, not serologic testing (*8*–*13*). One review of 22 studies from 10 countries estimated an overall household secondary attack rate of 17.1% (95% CI 13.7%–21.2%) (*8*), whereas another review found a pooled rate of 27% (95% CI 21%−32%) (*9*). Studies assessing the seroprevalence among close contacts, whether as a focus group or as part of national sample, have documented higher seroprevalences among close contacts than among persons who had not been in contact with patients who had suspected or confirmed COVID-19. A study in Singapore reported that 5.5% of household, 2.9% of work, and 2.1% of social contacts of COVID-19 patients were seropositive (*13*), whereas a study in Norway found that 31% of close contacts were seropositive (*14*). A large national serosurvey in England reported seroprevalences of 18.8% among those who had been in close contact with a confirmed COVID-19 patient and 16.9% among those who had been in contact with a suspected COVID-19 patient, compared with 4.3% among other participants (*15*). In addition, a nationwide population-based study in Spain reported seroprevalences of 31.4% among household members, 13.2% among noncohabitating family members or friends, and 10.6% among coworkers of COVID-19 patients (*16*). We assessed seroprevalence among close contacts of persons with COVID-19 in the Faroe Islands during the first wave of the pandemic in March and April 2020.

## Methods

### Data Collection

In this retrospective cohort study, we invited all close contacts of the 187 patients with confirmed COVID-19 in the Faroe Islands (crude prevalence 0.4%; https://corona.fo) during March 3–April 22, 2020. No local transmission occurred for the next 104 days, April 23–August 3, 2020 (*5*,*6*).

During this initial outbreak period, contact tracing was conducted by the Chief Medical Officer Office in the Faroes (Tórshavn, Faroe Islands), which communicated with all close contacts and requested that contacts quarantine for 14 days from the time of exposure. Close contacts of COVID-19 patients during the 48 hours before symptom onset, or of asymptomatic persons during the >48 hours before diagnosis, were traced. Household members and contacts who were within 2 meters of an infected person for >15 minutes, who had direct physical contact or provided caregiving without using personal protective equipment, or who had similar exposures, were defined as close contacts. 

All infected persons were asked to avoid contact with other persons in their household and to use separate bathrooms. Close contacts also were asked to self-quarantine and to avoid contact with other members of the household. Most patients and close contacts could successfully self-quarantine, except for those in households with small children. When patients or contacts were unable to adequately separate from household members, hotel rooms were offered free of charge by the government.

During their quarantine periods, close contacts were interviewed by telephone to monitor potential onset of symptoms (*5*). RT-PCR, which required a physician’s referral, was not used as a criterion for the end of quarantine. Only symptomatic contacts were tested for SARS-CoV-2 infection, although some asymptomatic contacts were also tested. Thus, no routine RT-PCR of all close contacts occurred during their quarantines.

The Chief Medical Officer’s Office emailed a participation request to all 854 close contacts of COVID-19 patients identified during March 3–April 22, 2020. Among the 854 close contacts, 132 had tested positive for SARS-CoV-2 infection by PCR; as a result, 722 contacts were eligible to participate in this study (Figure).

**Figure:**
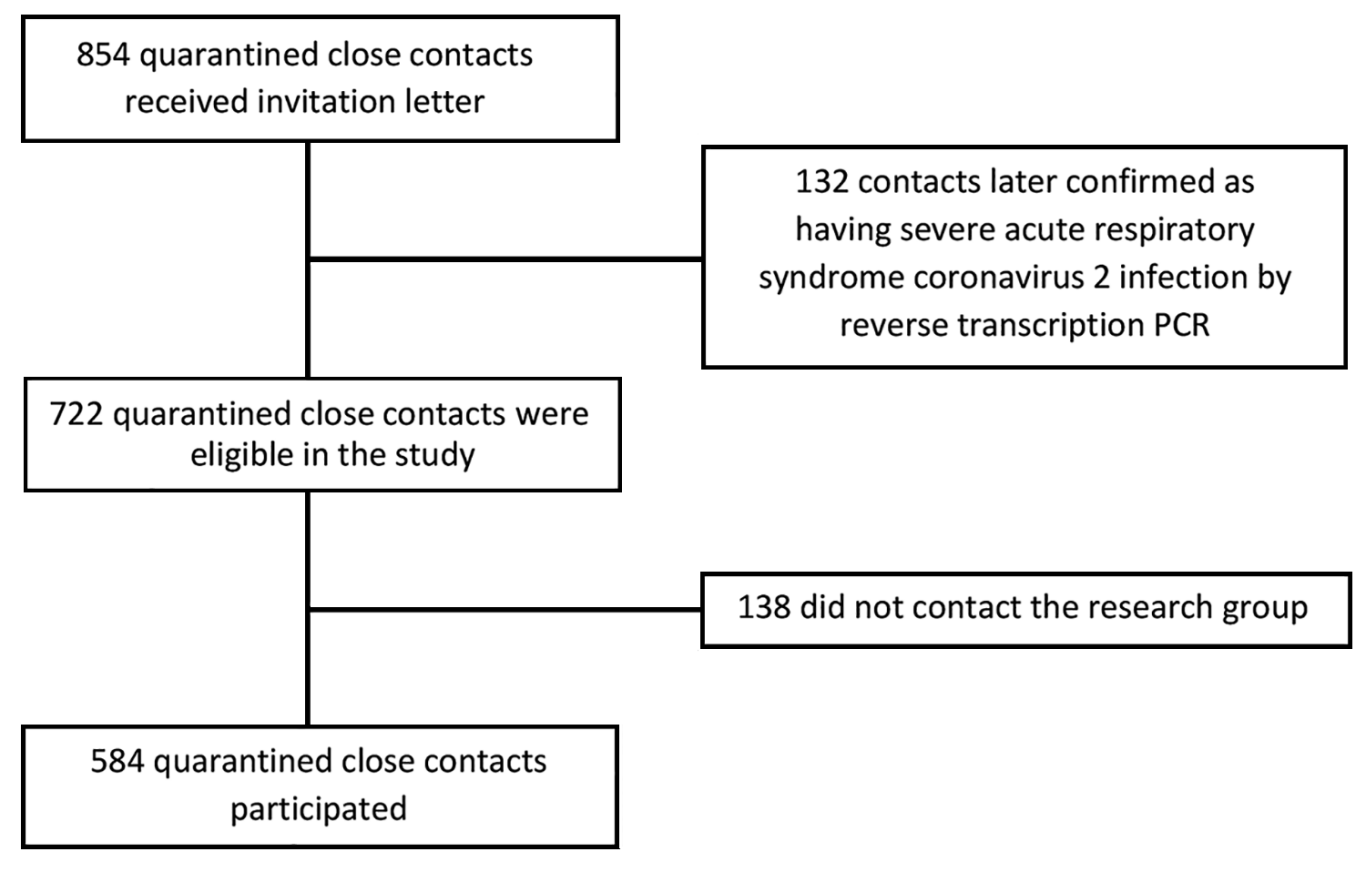
Recruitment of quarantined close contacts of coronavirus disease patients for study of seroprevalence of severe acute respiratory syndrome coronavirus 2–specific antibodies, Faroe Islands, 2020.

Participants gave informed consent to provide a blood sample and answer a short questionnaire. The questionnaire asked about demographic information, smoking habits, and medical history, as well as their experience in quarantine. For example, the questionnaire inquired whether participants had been tested by RT-PCR, whether they had experienced symptoms during quarantine, if other members of the household had also been quarantined, and whether they knew the identity of the infected person with whom they had contact. We used these data to classify all participants as household, workplace, or other (e.g., single event, social, or noncohabitating family) contacts. In total, 82 (14%) participants could not be classified on the basis of available data. We collected blood samples and administered questionnaires mainly during May 27–July 14, 2020; we also collected data from 3 participants earlier in May 2020 and 4 participants later in August and September 2020. No local cases were diagnosed in the Faroes during April 23–August 3, 2020. We telephoned seropositive participants about their results and asked them to recall symptoms experienced during the quarantine, which we registered alongside data from the telephone interviews conducted during quarantine. Parents responded on behalf of children <18 years of age. We used serum samples to determine patient serologic status by the commercially available Wantai SARS-CoV-2 Ab ELISA kit (Beijing Wantai Biologic Pharmacy Enterprise, http://www.ystwt.cn), according to the manufacturer’s instructions.

Informed consent was obtained from all participants. The study was approved by the Faroese Ethical Committee and the Data Protection Agency.

### Statistical Analysis

We estimated the crude seroprevalence by dividing the total number of seropositive cases by the total number of close contacts. We calculated categorical variables as percentages and continuous variables as means and SDs. We estimated 95% CIs for crude prevalence using exact binomial models. We adjusted for test performance as reported by independent evaluation (*17*) (sensitivity [96.7%, 95% CI 92.4%–98.6%] and specificity [99.5%, 95% CI 98.7%–99.8%]) by using bootstrap methods (*18*). To investigate possibly associated factors, we conducted regression analysis by using a binary outcome of seropositivity with the covariates of sex, age group (0–9, 10–17, 18–34, 35–49, 50–66, >67 years), history of smoking (ever/never), daily medication use (yes/no), chronic diseases (yes/no), and type of contact (household, workplace, other). We found that only contact type and age group were statistically significant; we included these covariates in the final model. We used SPSS Statistics 25.0 (IBM, https://www.ibm.com) for the analysis.

## Results

During March 3–April 22, 2020 in the Faroe Islands, 854 close contacts of COVID-19 patients were identified and quarantined, including 132 who were later confirmed to have SARS-CoV-2 infection. As a result, 722 close contacts that had not tested positive by RT-PCR were eligible for participation in this study (Figure); 584 participated, a participation rate of 80.9%. The mean participant age was 36 years (range 0–84 years), and 58% were women. Most participants were in the younger age groups; only 5.5% were >67 years of age (Table 1).

**Table 1:** Demographic and clinical characteristics of 584 quarantined close contacts of coronavirus disease patients in study of seroprevalence of SARS-CoV-2–specific antibodies, Faroe Islands, 2020*

Characteristic	Close contacts			p value†
	Total	Seronegative	Seropositive	
Total	584 (100.0)	552 (100.0)	32 (100.0)	
Sex				0.8
F	339 (58.0)	321 (58.2)	18 (56.3)	
M	245 (42.0)	231 (41.8)	14 (43.8)	
Mean age, y (SD) [range]	36.5 (20.2) [0.2–83.8]	37.0 (19.9) [0.2–81.7]	28.1 (24.0) [1.4–83.8]	0.02
Age group, y				<0.01
0–9	69 (11.8)	61 (11.1)	8 (25.0)	
10–17	68 (11.6)	62 (11.2)	6 (18.8)	
18–34	134 (22.9)	125 (22.6)	9 (28.1)	
35–49	131 (22.4)	128 (23.2)	3 (9.4)	
50–66	150 (25.7)	148 (26.8)	2 (6.3)	
>67	32 (5.5)	28 (5.1)	4 (12.5)	
Smoking status‡				0.1
Active	93 (16.7)	92 (17.4)	1 (3.6)	
Occasional	41 (7.3)	40 (7.5)	1 (3.6)	
Former	127 (22.8)	121 (22.8)	6 (21.4)	
Never	297 (53.2)	277 (52.3)	20 (71.4)	
Daily medication use§				0.6
Yes	145 (27.8)	137 (27.6)	8 (32.0)	
No	376 (72.2)	359 (72.4)	17 (68.0)	
Self-reported chronic diseases‡				0.06
Yes	278 (49.8)	268 (50.6)	10 (35.7)	
No	280 (50.2)	262 (49.4)	18 (64.3)	
Had PCR during quarantine¶				0.2
Yes	263 (48.3)	249 (47.8)	21 (65.6)	
No	281 (51.7)	272 (52.2)	11 (34.4)	
Had symptoms during quarantine#				0.7
Yes	116 (21.4)	112 (21.6)	14 (43.8)	
No	345 (63.5)	327 (63.1)	18 (56.3)	
Not sure	82 (15.1)	79 (15.3)	0	
Had other family members in quarantine**				0.2
No	120 (21.8)	118 (22.5)	2 (7.4)	
Yes, together	373 (67.7)	352 (67.2)	21 (77.8)	
Yes, separated	58 (10.5)	54 (10.3)	4 (14.8)	
Type of contact††				<0.01
Household	145 (28.9)	125 (26.5)	20 (64.5)	
Workplace	184 (36.7)	179 (38.0)	5 (16.1)	
Other	173 (34.5)	167 (35.5)	6 (19.4)	

*Values are no. (%) except as indicated. SARS-CoV-2, severe acute respiratory syndrome coronavirus 2. †p values determined by χ^2^ test for categorical variables or analysis of variance for continuous variables. ‡Questionnaire data missing for 26 quarantined close contacts, including 4 seropositive contacts. §Questionnaire data missing for 63 quarantined close contacts, including 7 seropositive contacts. ¶Questionnaire data missing for 40 quarantined close contacts, including 9 seropositive contacts. However, all seropositive patients were asked about PCR; as a result, values do not add up. #Questionnaire data missing for 41 quarantined close contacts, including 7 seropositive contacts. However, all seropositive patients were asked about symptoms; as a result, values do not add up. **Questionnaire data missing for 33 quarantined close contacts, including 5 seropositive contacts. ††Questionnaire data missing for 82 quarantined close contacts, including 1 seropositive contact.

A total of 32 (5.5% [exact binomial 95% CI 3.8%–7.7%]) persons, comprised of 17 women and 15 men who had not previously tested positive by RT-PCR, were seropositive for total antibody against SARS-CoV-2. After adjustment for test sensitivity and specificity, we calculated the prevalence of SARS-CoV-2–specific antibodies as 5.3% (95% CI 3.5%–7.5%). A total of 43.8% of the seropositive close contacts retrospectively reported symptoms, mainly fever, running nose, and loss of taste or smell. Most seropositive participants were in the youngest age group (0–9 years, mean 4.5 years, range 1.4–9.6 years) (Table 2). Among seropositive participants, 21 had received negative RT-PCR results during their quarantine; 11 participants recalled symptoms, whereas 10 did not. The other 11 seropositive participants did not have RT-PCR during quarantine, of whom 3 retrospectively recalled symptoms. Median time between quarantine and RT-PCR was 0 days (range –1 to 15 days); 11 participants had RT-PCR upon or before going into quarantine. RT-PCR was most prevalent among the youngest age group, of which all 8 participants had RT-PCR (Table 2). The presence of symptoms was not significantly different among the age groups (p = 0.9).

**Table 2:** Self-reported prevalence of symptoms and RT-PCR among 32 seropositive quarantined close contacts of coronavirus disease patients in study of seroprevalence of SARS-CoV-2–specific antibodies, by age, Faroe Islands, 2020*

Age, y	Total no. contacts	Had symptoms in quarantine	RT-PCR conducted	Mean days from start of quarantine to first RT-PCR (SD)	Median days from start of quarantine to first RT-PCR (range)
0–9	8	6 (75.0)	8 (100.0)†	5.5 (5.5)	6.0 (0–15)
10–17	6	0	4 (66.7)	7.0 (4.8)	8.5 (0–11)
18–34	9	5 (55.6)	3 (33.3)‡	0.3 (0.6)	0 (0–1)
35–49	3	1 (33.3)	3 (100.0)§	0.7 (2.1)	0 (–1 to 3)
50–66	2	1 (50.0)	1 (50.0)	0	0
>67	4	1 (25.0)	2 (50.0)	0	0

*Values are no. (%) except as indicated. RT-PCR, reverse transcription PCR; SARS-CoV-2, severe acute respiratory syndrome coronavirus 2. †Three contacts had 2 RT-PCRs. ‡One contact had 2 RT-PCRs and 1 had 4 RT-PCRs. §One contact had 2 RT-PCRs.

According to the multivariable logistic regression analysis, type of contact (p<0.01) and age group (p = 0.02) were significantly associated with seropositivity. The risk for seropositivity was significantly higher for household contacts compared with other contacts (adjusted odds ratio [aOR] 5.4, 95% CI 1.9–15.2). We did not find a statistically significant difference for workplace contacts compared with other contacts (p = 0.8). Overall, age was significantly associated with seropositivity (p = 0.02). Most age groups had lower aORs than the youngest age group (0–9 years), although these associations were statistically significant only for the 35–49-year (aOR 0.18, 95% CI 0.04–0.9) and 50–66-year (aOR 0.16, 95% CI 0.03–0.8) age groups. Participants >67 years of age had an increased aOR compared with the youngest age group; however, this association was not statistically significant (aOR 2.4, 95% CI 0.6–9.9). 

In this study, we identified 32 secondary SARS-CoV-2 infections by later serologic assay in addition to the 132 identified through initial RT-PCR, indicating a secondary attack rate of 19.2%. Most (67.5%) seropositive persons had been in quarantine with their families. In total, 65% of seropositive persons were infected by household members; when including noncohabitating close family members, this total rose to 71%. The other persons were infected by extended family, workplace, or social contacts. We identified 3 sibling pairs, 2 sets of spouses, and 3 parent–child pairs among the seropositive persons.

## Discussion

In this retrospective cohort study of close contacts of COVID-19 patients, we found a 5.5% seropositivity rate among contacts who were not previously identified as positive by RT-PCR. Seroprevalence was highest among household contacts. We found the highest seropositivity rate among children who were infected by their parents. The risk for seropositivity among household contacts was 5-fold that of the risk posed by other close contacts, probably because household members might have closer and more prolonged interactions than work or social contacts. In total, 56% of secondary infections were asymptomatic.

The rapid spread of COVID-19 is partly attributable to transmission by asymptomatic or presymptomatic persons; many of these cases remain undetected because patients might not seek healthcare or undergo testing (*19*). As a result of the large-scale testing and tracing protocols used in the Faroe Islands, the risk for transmission from persons with undetected cases is probably low. A study of 1,075 randomly selected persons from the Faroe Islands in April 2020 found a 0.6% seropositivity rate (exact binomial 95% CI 0.2%–1.2%), which corresponds to 313 COVID-19 patients (*2*), somewhat higher than the observed 0.4% crude prevalence of confirmed cases in the Faroe Islands [https://corona.fo]). Our results for close contacts of patients with confirmed COVID-19 are in accordance with the seroprevalence study (*2*) indicating the existence of some undetected (i.e., not documented in the official records) cases of SARS-CoV-2 infection in the Faroe Islands. Our results highlight the importance of tracing close contacts, who have a much higher seroprevalence than the general population, despite a high proportion of asymptomatic seropositive persons. These findings underscore that testing only symptomatic contacts will miss infections and underestimate the true number of cases. In addition, we emphasize that a negative RT-PCR result might not rule out SARS-CoV-2 infection in a household contact. The close contacts who underwent RT-PCR were tested at a median 0 days (range −1 to 15 days) from start of quarantine (Table 2), possibly indicating that most patients were tested before symptoms developed. Because most of the seropositive contacts in this study were infected by household members, our findings emphasize the importance of isolating infected persons. 

Our overall prevalence estimate is lower than that of a study comprising 100,000 participants in England, which found an 18.8% (742/3,946) seroprevalence among those who had unspecified contact with a COVID-19 patient (*15*). Our estimate is more comparable with that of a study in Singapore that found a 5.5% (29/524) seroprevalence among household contacts who did not have a COVID-19 diagnosis (*13*). In a seroprevalence study of household members of COVID-19 outpatients in Norway, Cox et al. (*14*) found that 24/77 (31%) household members were seropositive 6 weeks after the index patient had first tested positive by RT-PCR. Similarly, a study of 61,075 participants in Spain found a 10.6% seroprevalence among coworkers, compared with 31.4% among household members of COVID-19 patients (*16*). These prevalence estimates are higher than our overall prevalence of 5.5% among close contacts who were not previously identified as positive by RT-PCR. However, comparing seroprevalence studies can be challenging because of differences in the nature and closeness of contacts, classification of contact types, methods of measuring antibodies, and characteristics of eligible study participants. In addition, the level of community transmission in each country would affect prevalence.

Among the 32 seropositive persons we identified, 21 had tested negative by RT-PCR, including 11 (52.4%) who had reported symptoms. The sensitivity of RT-PCR is dependent on multiple factors, primarily the presence of viral RNA on the test swab, which can vary by swabbing technique and whether viral RNA is present at the anatomic site of the test. Therefore, the timing of testing in relation to infection onset is critical. Among participants who underwent RT-PCR, most were tested during the early stage of quarantine, probably before symptom onset, and thus perhaps also before infection onset (e.g., among contacts who were in quarantine with the infected family member), which might explain their negative RT-PCR results (Table 2). In addition, many of the quarantined close contacts who were tested by RT-PCR were young children, in whom collecting adequate swab samples might be difficult.

The major strengths of this study are its nationwide nature, in which all close contacts of confirmed COVID-19 patients from the first wave in the Faroe Islands were directly contacted, and the high participation rate. As a result, the likelihood of selection bias is low. Because the Faroe Islands eliminated COVID-19 for 104 days after the last patient in this wave was identified, there was little risk for exposure from sources other than the index cases. 

One limitation of our study is that contacts were asked retrospectively about symptoms during quarantine, introducing the possibility for recall bias. The risk for bias is especially relevant for children, whose questionnaires were answered by parents. However, because most index cases occurred in a family member, parents were probably vigilant for potential symptoms in children. Another limitation might be the classification of participants as household, workplace, or other contacts. Because this classification was based on whether participants knew the identity of the index patient, this measure might be imprecise. However, we were able to classify most participants into 3 groups on the basis of available information. We found that the primary exposure route is within families, and we believe this information is valuable, even if the data might be flawed. Finally, RT-PCR was not routinely conducted for quarantined persons. Because symptomatic persons were probably prioritized for RT-PCR, this selection might have introduced bias.

In conclusion, our study found that seroprevalence among close contacts of COVID-19 patients is higher than that among the general population. Close contacts, especially household members, of COVID-19 patients are at higher risk for infection. Thus, routinely testing household contacts of COVID-19 patients, regardless of symptoms, might improve detection of SARS-CoV-2 infection. Our results also indicate that close contacts should maintain quarantine even if they receive negative RT-PCR results early in quarantine.
